# Why Students Do Not Engage in Contract Cheating

**DOI:** 10.3389/fpsyg.2019.02229

**Published:** 2019-10-04

**Authors:** Kiata Rundle, Guy J. Curtis, Joseph Clare

**Affiliations:** ^1^Discipline of Psychology, Murdoch University, Perth, WA, Australia; ^2^School of Psychological Science, University of Western Australia, Perth, WA, Australia; ^3^School of Law, University of Western Australia, Perth, WA, Australia; ^4^School of Law, Murdoch University, Perth, WA, Australia

**Keywords:** contract cheating, ghostwriting, academic misconduct, university students, college students

## Abstract

Contract cheating refers to students paying a third party to complete university assessments for them. Although opportunities for commercial contract cheating are widely available in the form of essay mills, only about 3% of students engage in this behaviour. This study examined the reasons why most students do *not* engage in contract cheating. Students (*n* = 1204) completed a survey on why they do not engage in contract cheating as well as measures of several individual differences, including self-control, grit and the Dark Triad traits. Morality and motivation for learning received the greatest endorsement for why students do not engage in contract cheating. Controlling for gender, individual differences predicted students’ reasons for not contract cheating. This study supports the use of criminological theories relating to rational choice, self-control and opportunity to explain why students do not engage in contract cheating. Practically, this study may inform academic policies and assessment design that may reduce contract cheating.

## Introduction

Clarke and Lancaster (2006) first defined contract cheating as when a student pays a ghostwriter to complete an assessment for them, with the inclusion of a financial exchange. This definition distinguishes contract cheating as an independent form of academic misconduct, despite its similarities with ghostwriting *per se*. Since this seminal work, contract cheating has gained notoriety as a problem facing tertiary institutions around the world (e.g., Cook, 2017; Vallance, 2018) and there has been substantial recent research examining contract cheating and other outsourcing behaviours (e.g., Walker and Townley, 2012; Rigby et al., 2015; Clare et al., 2017; Curtis and Clare, 2017; Ellis et al., 2018; Rowland et al., 2018; Bretag et al., 2019). In higher education, assessment exists as a measure of learning, and academic misconduct, such as cheating, undermines the validity of higher education assessments. Contract cheating services, often referred to as essay mills, are highly accessible to students through simple internet searches (e.g., Australian Help, 2018; Best Essays, 2018; Essay Roo, 2018). Despite the ease of potential engagement in contract cheating, the prevalence of this behaviour is low [e.g., Curtis and Vardanega (2016), estimated only 2.8–3.4% of students engaged in contract cheating and Newton (2018) estimated 3.5% of students from around the world from 1978 to 2016 engaged in contract cheating]. This paper uses a range of theoretical perspectives that attempt to explain rule-breaking behaviour in other contexts to examine why the majority of students *do not* engage in contract cheating: a question that remains unanswered by research to date.

The rational choice perspective (Cornish and Clarke, 1987) states that criminal and unethical behaviour is not necessarily objectively rational, but it is driven by people’s capacity to rationalise their actions. Thus, to understand ethical and unethical behaviour, it is important to examine people’s rationalisations or *reasons* for their behavioural choices. In this paper we propose that other criminological theories and psychological individual differences will be related to the extent to which students endorse various reasons for not engaging in contract cheating. The remainder of the introduction briefly summarises the relevant theoretical perspectives, including the General Theory of Crime (Gottfredson and Hirschi, 1990), routine activity theory (Cohen and Felson, 1979), and a range of personality-based individual-differences as explanations for academic misconduct. As is explained, seven key themes emerge from the literature to underpin the reasons why the vast majority of students do not engage in contract cheating: (1) opportunity, (2) fear of detection and punishment, (3) trust, (4) motivation for learning, (5) time management, (6) morals, and (7) norms.

The General Theory of Crime (Gottfredson and Hirschi, 1990) proposes that a lack of self-control is the foundation of all crime. Self-control is defined as the ability to regulate one’s own behaviour and impulses (Tangney et al., 2004). Gottfredson and Hirschi (1990) suggested that individuals with low self-control are more likely to engage in criminal behaviour as they are more susceptible to temptation and the *opportunity* therein. As a distinctly fraudulent, self-interested behaviour (Walker and Townley, 2012), contract cheating fits into the definition of crime provided within the General Theory of Crime, which was developed to encompass acts that are analogous with crime (Gottfredson and Hirschi, 1990). Muraven et al. (2006) proposed that self-control exists as a reservoir that can be depleted by demands, such as stress. This concept suggests that anyone can have a failure in self-control, resulting in an increased susceptibility to temptation, as opposed to only people with low self-control being susceptible, as per the General Theory of Crime. Consequently, this means that regardless of a student’s self-control, given the opportunity, it is possible for any student to succumb to the temptation of contract cheating. The converse of this theory is that if a student is able to maintain their self-control reservoir, then they are less likely to be susceptible to the temptation to cheat. This is supported by Gino et al. (2011), who found that individuals who experience a depletion of self-control are more likely to engage in unethical behaviours, such as cheating, compared to those who do not experience this depletion. Having higher trait self-control would thus be advantageous as it would take a greater number of stressors to deplete the individual’s reservoir. The theoretical platform of self-control has been used in numerous studies on academic misconduct to describe and explain student engagement in plagiarism (see Cochran et al., 1998; Curtis et al., 2018). Both Cochran et al. (1998) and Curtis et al. (2018) found higher levels of self-control were protective against student engagement in cheating behaviours.

In addition to the importance that The General Theory of Crime places on opportunity, routine activity theory (Cohen and Felson, 1979) and the rational choice perspective (Cornish and Clarke, 1987) are alternative criminological theories that also explain the important role opportunity plays in the non-random nature of crime across time and place. Routine activity theory proposes that for crime to occur a motivated offender, suitable target or victim, and the absence of a capable guardian (person or thing that acts to protect a target from crime) must co-occur in time and space (Cohen and Felson, 1979). In the context of contract cheating, this theory requires a student to be motivated to purchase a university assessment to submit as their own, this may include succumbing to the temptation as a result of a failure of their self-control (discussed above); that the student has a suitable assignment to contract; and that they are able to do so without the supervision of a guardian. With a constant stream of assignments to complete throughout each semester, and an equivalent guardianship issue in each case due to online submission, and no systematic way of detecting contract cheating, students do not have a shortage of potential opportunities.

The rational choice perspective emphasises that people weigh the risk-reward-effort rationale when deciding whether to act unethically (Cornish and Clarke, 1987), e.g., does receiving a better grade outweigh the fear of detection and punishment? Being unable to rationalise the decision and perceiving the risks as outweighing the benefits would thus explain why students do *not* engage in contract cheating. Ogilvie and Stewart’s (2010) findings support this explanation, as students who did not perceive plagiarism behaviours as beneficial and who believed they would experience shame from engaging in the activity were less likely to engage in plagiarism.

The risk-reward model of the rational choice perspective is also believed to be utilised in *trust* (Mayer et al., 1995). Evans et al. (2011) proposed that trust is subject to the situation and cognitive ability of the individual. A concept that is consistent with the rational choice perspective. Mayer et al. (1995) noted that a trustor must be willing to put themselves in a vulnerable position in order to trust a third party to complete certain actions. The idea that trust is a rational decision made by an individual, at least in the context of contract cheating, suggests that for students to trust an essay mill or other contracted service, they must accept both the risks and potential rewards. Students must trust that the contracted service will not only complete their assessment but also meet their assessment requirements, including word length and deadline, and that the benefit of engaging in contract cheating, such as better grades, outweighs the risk of detection and subsequent punishment. Moreover, these risks include students being unable to verify the originality or quality of a paper purchased through an essay mill (Rigby et al., 2015). Students risk receiving a paper that has been submitted previously by another student, which may result in detection via text-matching software. Consequently, students who do not trust a contracted third party to complete their assessment to their desired standards may be unable to rationalise the decision to engage in contract cheating and, furthermore, may believe they are more competent than the service.

The General Theory of Crime, routine activity theory, and the rational choice perspective all propose that anyone is capable of engaging in deviant behaviour, such as contract cheating. Although these theories focus on *why* someone engages in a socially undesirable behaviour, they also offer insight into the *why not*. Having high self-control, not having the necessary opportunity structure, and being unable to rationalise the behaviour or trust a third party are all potential explanations of why students do *not* engage in contract cheating. Although these theories provide a good theoretical understanding, we can learn more about why students do *not* engage in contract cheating through an empirical perspective.

Students have varying *motivations* for attending university. Blum (2016) notes a “tension between ‘learning for learning’s sake”’ and a “getting through it” attitude (p. 392), such as that passing is sufficient to graduate, may lead students to engage in cheating behaviours. Park (2003) proposed that efficiency gain (i.e., obtaining a better grade in less time and with less effort) may contribute to students’ decisions to cheat. Students are under immense pressure to perform well at university (Devlin and Gray, 2007) with grades affecting future learning and employment opportunities, and scholarship eligibility (Walker and Townley, 2012). Issues with *time management* throughout the semester can exacerbate this pressure as desperate students may feel they have inadequate time to complete their assessments, and thus turn to purchasing assignments (Franklyn-Stokes and Newstead, 1995). Franklyn-Stokes and Newstead (1995) reported that 55% of respondents listed time pressures as a reason for “submitting coursework from an outside source” (p. 171) such as buying essays from a former student or essay mill. Additionally, short turnaround times on assessments increase the likelihood of student engagement in contract cheating (Bretag et al., 2018b). These pressures may push students to succumb to the temptation to engage in contract cheating as a potentially effective and efficient method to increase their performance, with some essay mills allowing students to select their desired grade for the contracted assignment (see The Uni Tutor, 2017). However, there is inconsistent support in the literature for the role of grades as a predictor of academic misconduct (Ogilvie and Stewart, 2010; Hensley et al., 2013; Kuntz and Butler, 2014). Franklyn-Stokes and Newstead (1995) found that students reported that purchasing assignments would devalue the sense of achievement they obtained from completing assessments themselves. This sense of achievement is likely associated with the satisfaction of the psychological need for competence (Longo et al., 2016). Competence is defined as the feeling of effectiveness individuals experience upon completing a task or activity to their desired outcome (Longo et al., 2016). Ogilvie and Stewart (2010) found that students who do not feel competent are unlikely to complete their assessments. This may further drive students toward the temptation to engage in contract cheating. Additionally, academic ability has been found to be driven by self-control and grit (Tangney et al., 2004; Duckworth and Quinn, 2009). Gritty individuals are able to maintain their focus and passion for long-term goals, even in the absence of positive reinforcement (Duckworth and Quinn, 2009). Self-control and grit are strongly correlated and have both been associated with high academic performance (Tangney et al., 2004; Duckworth and Quinn, 2009) and success in life (Duckworth and Gross, 2014). Although the concept of grit has recently been criticised (Credé et al., 2017), recent evidence in the contract cheating literature suggests that a lack of perseverance is a critical factor in students’ decision to cheat (Amigud and Lancaster, 2019).

Personality traits are regularly identified in the literature as predictors of why students engage in academic misconduct (see Nathanson et al., 2006; Williams et al., 2010; Lewis and Zhong, 2011; Wilks et al., 2016). Nathanson et al. (2006) and Williams et al. (2010) found that the Dark Triad traits of Machiavellianism, narcissism, and psychopathy (Jones and Paulhus, 2011) are a better predictor of student engagement in academic misconduct when compared to the Big Five personality traits of openness, conscientiousness, extraversion, agreeableness, and emotional stability. This may result from individuals with Dark Triad personality traits possessing a greater willingness to engage in norm-violating behaviours (Muris et al., 2017). However, the Dark Triad traits have been positively associated with self-monitoring (Kowalski et al., 2018), meaning individuals with these traits use situational cues to determine appropriate social behaviour (Snyder, 1974). Thus, despite being a predictor of academic misconduct, students who score highly on measures of the Dark Triad traits may not engage in contract cheating due to situational cues, such as apparent risks and punishments, even if they are not dissuaded from contract cheating by moral or normative reasons.

Along with grades and personality, *morals* and *norms* also appear in the literature on academic misconduct (Park, 2003; Stephens, 2018) and have strong ties with each other (Posner and Rasmusen, 1999). Franklyn-Stokes and Newstead (1995) noted that students predominantly did not engage in assignment purchasing due to the perception of the behaviour as immoral and dishonest. Moss et al. (2018) found that strong moral virtues are negatively associated with engagement in plagiarism. This perception of morality aligns with injunctive norms (i.e., the belief of what constitutes acceptable social behaviour in a given situation, Locke et al., 2017), which condemn academic misconduct, including contract cheating. Additionally, acting in opposition of norms is believed to have a psychological cost, such as guilt (Locke et al., 2017). By not engaging in contract cheating, students are aligning their moral values with the injunctive norms and subsequently avoiding the psychological costs. However, Selwyn (2008) found that plagiarism is often perceived by students as an accepted and even expected aspect of student life. This perception of the descriptive norms (i.e., the belief of what others would do in the same situation, Locke et al., 2017), is believed to encourage other students to engage in similar behaviours (Davis et al., 1992; Jurdi et al., 2012). Students may rationalise their engagement in plagiarism by proclaiming that they plagiarise less than their peers (Hale, 1987, as cited in Moss et al., 2018). Thus, students’ perceptions of peer engagement in contract cheating may influence their own decision to engage in contract cheating. Bretag et al. (2018a) asked students to estimate the prevalence of contract cheating amongst their peers, responses ranged across the board, from rates of 0 to 91–100% of students. The largest subgroup of the sample (22.1% of respondents) estimated prevalence to fall between 1 and 10% (Bretag et al., 2018a). The varied perceptions of peer engagement in contract cheating is likely to affect students’ perceptions of the descriptive norms, subsequently impacting upon their decision of whether to engage in contract cheating.

Student engagement in contract cheating is low, despite available opportunities. The factors discussed above, including student motivation for learning, provide potential explanations for why students do *not* engage in contract cheating. Understanding how these factors combine with personality-based individual-differences along with the application of criminological theories can help establish the reasons why the majority of students refrain from contract cheating. Given the lack of previous similar research, the current study had the exploratory aim of investigating the reasons why students do not engage in contract cheating and to see what capacity criminological theories and psychological constructs have to explain and influence these reasons. Using an online survey, university students were asked to indicate their extent of agreement with various reasons why they do not engage in contract cheating and to complete measures of individual differences examining self-control, competence, grit, and personality. Stage One of the data analysis explored whether the predicted seven themes were present in the Reasons for Not Contract Cheating (RNCC) measure (see section “Materials and Methods”). Drawing on the relevant research findings outlined above, Stage Two of the data analysis tested the following hypotheses.

*H1.* Self-control will negatively relate with a lack of opportunity as a reason for not engaging in contract cheating.

*H2.* The psychological constructs of self-control, satisfaction of students’ need for competence, and grit will positively relate to students’ motivation for learning as reasons for not engaging in contract cheating.

*H3.* The Dark Triad personality traits of Machiavellianism, narcissism, and psychopathy will be negatively related with morals and norms as reasons for not engaging in contract cheating.

*H4.* The Dark Triad personality traits will positively relate with fear of detection and punishment as reasons for not engaging in contract cheating.

## Materials and Methods

### Participants and Design

A sample of 1808 university students was recruited online through a Western Australian University’s Research Participation Portal^1^, SurveyCircle (an online survey sharing website), and social media – principally university student Facebook groups. Given the focus of this research on non-cheaters, any responses where past engagement in contract cheating was indicated (*n* = 27 [2%]) or not disclosed (*n* = 14) were removed from analysis. Other exclusions were made based on: failure to respond to any questions (*n* = 42), not being currently enrolled in a degree or course (*n* = 167), and responses that lacked variation (*n* = 4, e.g., responding *strongly disagree* to all items). Participants under 18 years old were also excluded (*n* = 8). A further 342 cases were removed that had more than 10% missing data. This resulted in a total of 604 response exclusions.

The final sample of 1204 consisted of undergraduate and postgraduate students from around the world, including Australia and New Zealand, the United Kingdom, several African countries, the United States, Canada, and numerous South American, European, and Asian countries. Over 55% of participants were Australian. Participants were aged between 18 and 59 (*M* = 24.77, *SD* = 7.39, *median* = 22). The sample consisted predominantly of female students (87.5%), only 10.1% of participants identified as male, a further 32 (2.7%) students did not report their gender or selected the response option *not specified*. Just over 20% of participants reported completing a psychology major and 17.9% reported attending the Western Australian University.^2^

### Materials and Measures

#### Reasons for Not Contract Cheating Measure

A 21-item measure of the reasons why students do not engage in contract cheating was developed for this study, referred to as the RNCC measure. Items were developed by the authors, and reviewed by a focus group of senior students, based on the seven themes identified in the literature: opportunity, fear of detection and punishment, trust, motivation for learning, time management, morals, and norms. Contract cheating was defined as the process of a student paying a third party to complete a university assessment on their behalf and claiming the work as their own, including: written assignments, exams, online tests, and other assessment items. Participants were asked to indicate the extent to which an item contributed to the reasons they do not engage in contract cheating, using a five-point Likert scale, where 1 = *not a reason at all* and 5 = *this is the main reason I do not*. All items for this measure are presented in Table 1 and the Appendix. Participants were asked if there were any other reasons that contributed to why they do not engage in contract cheating.

**TABLE 1:** Item descriptives and loadings on five factors with orthogonal rotation.

			**Factors**				
**Item**	***M***	***SD***	**1**	**2**	**3**	**4**	**5**
I am afraid of being punished	2.97	1.39	0.899	–	–	–	–
I am afraid of getting caught	3.05	1.41	0.872	–	–	–	–
I’m afraid that being caught would have long-term career implications	3.37	1.44	0.738	–	–	–	–
I am worried I would be caught by plagiarism software like Turnitin or Urkund	2.79	1.47	0.669	–	–	–	–
I’m afraid that if others found out I would be judged negatively or ridiculed	2.54	1.44	0.535	–	–	–	–
I don’t trust people to do my assignment well	2.93	1.49	–	0.912	–	–	–
I don’t trust people to do my assignment on time	2.34	1.47	–	0.727	–	–	–
I feel I could do better than someone I paid	2.67	1.49	–	0.476	–	–	–
I manage my time well and don’t have to resort to cheating^∗^	2.85	1.44	–	0.352	0.333	–	–
I would feel shame, guilt or remorse	3.77	1.24	–	–	0.618	–	–
I wouldn’t do it because I would not want others to do it	3.04	1.45	–	–	0.613	–	–
I think it is wrong or immoral	4.20	1.01	–	–	0.564	–	–
I could not find someone to write on the specific assignment topic	1.29	0.78	–	–	–	0.597	–
I would not know where to find someone to write an assignment for me	2.09	1.31	–	–	–	0.543	–
I can’t afford to pay someone to write assignments for me	2.00	1.32	–	–	–	0.491	–
I have not completed any assignments, so I have not had the chance or need	1.13	0.58	–	–	–	0.466	–
I don’t care enough about grades to want to cheat to do better	1.38	0.87	–	–	–	0.396	–
I don’t think other students like me would pay someone to write their assignment^∗^	2.13	1.33	–	–	0.349	0.353	–
I feel I’d only be cheating myself because I am studying to learn	3.83	1.23	–	–	–	–	0.694
I am studying to learn rather than to get a qualification/degree	3.20	1.34	–	–	–	–	0.649
I want the sense of achievement from doing work myself^∗^	3.86	1.18	–	0.448	–	–	0.450

*Eigenvalues > 1. ^∗^Item removed from factor after CFA.*

#### Brief Self-Control Scale

Self-control was measured using the Brief Self-Control Scale (Tangney et al., 2004). Participants were asked to respond how representative each item was of them using a five-point Likert scale, where 1 = *not at all* and 5 = *very much*. The 13-item measure included four non-reversed items, such as “I am good at resisting temptation,” and nine reversed items. This scale demonstrated acceptable fit in a CFA as a single-factor measure (χ^2^[51] = 244.86, *p* < 0.001, GFI = 0.968, CFI = 0.958, TLI = 0.936, RMSEA = 0.056 [0.049, 0.063]).

#### Need Satisfaction and Frustration Scale – Competence Subscale

The Need Satisfaction and Frustration competence subscales were used to measure how effective and capable students feel when completing tasks and mastering challenges (Longo et al., 2016). Participants were asked to respond to the six-items in relation to their studies using a seven-point Likert scale, where 1 = *strongly disagree* and 7 = *strongly agree*. The measure included three competence frustration items, such as “I doubt whether I am able to carry out my tasks properly,” and three competence satisfaction items, such as “I feel I am very good at the things I do.” This scale demonstrated good fit in a CFA as a two-factor measure (χ^2^[6] = 50.82, *p* < 0.001, GFI = 0.986, CFI = 0.986, TLI = 0.965, RMSEA = 0.079 [0.060, 0.099]).

#### Short Grit Scale

Grit was measured using the Short Grit Scale (Duckworth and Quinn, 2009). The measure has two subscales: consistency of interest and perseverance of effort. Each subscale consists of four-items, including: “I often set a goal but later choose to pursue a different one” (reversed) for consistency of interest, and “I finish whatever I begin” for perseverance of effort. Participants were asked to indicate how like them each statement is on a five-point Likert scale, where 1 = *not at all like me* and 5 = *very much like me*.^3^ This scale demonstrated good fit in a CFA as a two-factor measure (χ^2^[17] = 75.35, *p* < 0.001, GFI = 0.984, CFI = 0.983, TLI = 0.972, RMSEA = 0.053 [0.041, 0.066]).

#### Dark Triad Dirty Dozen

Personality was assessed using the Dark Triad Dirty Dozen, a 12-item measure of Machiavellianism, narcissism, and psychopathy (Jonason and Webster, 2010). Machiavellianism items included, “I have used deceit or lied to get my way”; narcissism items included, “I tend to want others to admire me”; and psychopathy items included, “I tend to lack remorse.” Participants responded to the measure on a nine-point Likert scale, where 1 = *strongly disagree* and 9 = *strongly agree*. This scale demonstrated acceptable fit in a CFA as a three-factor measure (χ^2^[46] = 352.64, *p* < 0.001, GFI = 0.951, CFI = 0.962, TLI = 0.945, RMSEA = 0.074 [0.067, 0.082]).

#### Demographics

Participants were asked to respond to a series of demographic questions, including: age, gender, institution, university major, and country.

### Procedure

The first page of the online survey was a consent and information letter. Consent was implied by progressing past this page. Participants were then asked if they were currently enrolled in a university degree or course, *no* responses were directed to the end of the survey. *Yes* respondents progressed to the next page where they were provided with the definition and asked if they had ever engaged in contract cheating (see Appendix). *Yes* responses to this question were redirected to a separate survey, whilst *no* responses progressed. Participants were required to respond to the first two questions to progress through the rest of the survey.

Participants who had not engaged in contract cheating were presented with the RNCC measure, followed by the psychological measures in the following order: Dark Triad Dirty Dozen, Brief Self-Control Scale, Need Satisfaction and Frustration competence subscales, and Short Grit Scale, then the demographic questions. The order of the measures was fixed due to expected attrition. Responses to the measures were not compulsory.

The survey took approximately 15 min to complete and had to be completed in a single sitting. After completing the survey, participants were thanked and offered a choice of the three available rewards.

## Results

### Data Screening and Assumption Testing

Scale means were calculated for each of the psychological measures’ subscales, with items reversed where necessary. Means and standard deviations are presented in Table 2. The data were screened for violations of statistical assumptions prior to testing. Cronbach’s alpha internal consistency reliability analyses were calculated for the measures, with results reported on the diagonal in Table 2. Less than 0.3% of data were missing and Little’s MCAR test showed that these data were missing completely at random, χ^2^(1552) = 1573.76, *p* = 0.344; missing scores were imputed for analyses.

**TABLE 2:** Cronbach’s alpha internal consistency reliability and correlations between the five factors and psychological measures.

	***M (SD)***	**1**	**2**	**3**	**4**	**5**	**6**	**7**	**8**	**9**	**10**	**11**	**12**	**13**
**Factors**														
(1) Fear of punishment and detection	2.95(1.18)c	(0.88)												
(2) Self-efficacy and mistrust	2.65(1.22)d	0.33^∗^	(0.76)											
(3) Morality and norms	3.67(0.95)a	0.19^∗^	0.10	(0.64)										
(4) Lack of opportunity	1.58(0.65)e	0.41^∗^	0.34^∗^	–0.07	(0.65)									
(5) Motivation for learning	3.52(1.13)b	−0.14^∗^	0.10	0.36^∗^	−0.17^∗^	(0.72)								
**Psychological measures**														
(6) Machiavellianism	3.13 (1.84)	0.19^∗^	0.12^∗^	−0.22^∗^	0.17^∗^	−0.23^∗^	(0.87)							
(7) Narcissism	3.91 (1.92)	0.20^∗^	0.16^∗^	–0.09	0.13^∗^	−0.17^∗^	0.63^∗^	(0.86)						
(8) Psychopathy	2.49 (1.51)	0.09^∗^	0.11^∗^	−0.26^∗^	0.20^∗^	−0.20^∗^	0.60^∗^	0.46^∗^	(0.78)					
(9) Self-control	3.06 (0.70)	−0.13^∗^	0.08^∗^	0.23^∗^	−0.23^∗^	0.25^∗^	−0.43^∗^	−0.35^∗^	−0.39^∗^	(0.86)				
(10) Competence satisfaction	4.79 (1.17)	–0.08	0.21^∗^	0.11^∗^	−0.14^∗^	0.16^∗^	–0.06	0.00	–0.09	0.40^∗^	(0.86)			
(11) Competence frustration	4.30 (1.33)	0.13^∗^	0.09	–0.06	0.16^∗^	−0.11^∗^	0.17^∗^	0.14^∗^	0.13^∗^	−0.44^∗^	−0.59^∗^	(0.77)		
(12) Consistency of interest	2.97 (1.00)	0.16^∗^	0.01	0.11.^∗^	0.20^∗^	−0.19^∗^	0.36^∗^	0.34^∗^	0.34^∗^	−0.60^∗^	−0.24^∗^	0.34^∗^	(0.86)	
(13) Perseverance of effort	3.47 (0.72)	–0.08	0.14^∗^	0.20^∗^	−0.14^∗^	0.24^∗^	−0.21^∗^	−0.14^∗^	−0.23^∗^	0.58^∗^	0.46^∗^	−0.38^∗^	−0.49^∗^	(0.70)
Gender	1.90 (0.30)	–0.02	–0.02	0.17^∗^	–0.06	0.07	−0.13^∗^	−0.15^∗^	−0.26^∗^	0.08	–0.03	0.04	–0.08	0.07

**N* = 1204 except for gender correlations where *N* = 1173, ^∗^*p* < 0.001 (two-tailed). Cronbach’s alpha internal consistency reliabilities are on the diagonal in parentheses. Subscript a > b > c > d > e, *p* < 0.05. Gender was coded as male = 1, female = 2.*

### Stage One: Factor Analysis

A principal components analysis with oblimin (oblique) rotation was conducted on the 21-items of the RNCC measure. This analysis was run to assess the assumptions of whether a factor analysis with orthogonal rotation was appropriate. The sample size exceeded 1000 participants, which is excellent (Tabachnick and Fidell, 1996). Sample adequacy was further verified by the Kaiser-Meyer-Olkin measure (*KMO* = 0.84). A visual inspection of the correlation matrix, scree plot, and item loadings with oblimin rotation indicated that the items were factorable. The identified factors had inter-correlations less than 0.30, which support the use of a principal axis factor analysis with orthogonal rotation (Tabachnick and Fidell, 1996).

A principal axis factor analysis with varimax (orthogonal) rotation was then conducted on the 21-items. This analysis returned five factors with eigenvalues exceeding 1, these factors accounted for 48.62% of the total variance. The five factors were labelled based upon the themes present in the loaded items, these are: (1) Fear of Detection and Punishment, (2) Self-Efficacy and Mistrust, (3) Morality and Norms, (4) Lack of Opportunity, and (5) Motivation for Learning. Item descriptives and rotated loadings can be seen in Table 1.

As Table 1 indicates, three items had similar loadings on two factors. To confirm the factor structure of the RNCC measure, we conducted CFA in AMOS 25.0. The initial analysis suggested sub-optimal model fit (χ^2^[179] = 1812.04, *p* < 0.001, GFI = 0.863, CFI = 0.827, TLI = 0.797, RMSEA = 0.087 [0.083, 0.091]). Modification indices (MI) and standardised residuals were examined, and a model with reasonable fit was achieved by removing the three items with ambiguous factor loading and co-varying error terms on items within the same factor when MI’s > 15; χ^2^(116) = 653.99, *p* < 0.001, GFI = 0.935, CFI = 0.932, TLI = 0.921, RMSEA = 0.062 (0.058, 0.067). Notably, as can be seen in Table 1, one of the items removed (“I want the sense of achievement from doing work myself”) had the second-highest mean endorsement by students as a reason for not engaging in contract cheating, and this finding may be of interest to practitioners.

The mean of the items in each factor were calculated and are presented in Table 2. Cronbach’s alpha coefficients were calculated for the five factors to test their internal consistency (see Table 2). Two factors, Morality and Norms and Lack of Opportunity, returned coefficient scores below 0.70. The item-total statistics showed that removal of any of the items in these two factors would result in a reduction of the alpha coefficient. These factors appear, based on the item wording, to be conceptually meaningful groups of questions that were distinct from the other items in the RNCC scale. For these reasons, no further items were removed.

A repeated measures ANOVA was conducted to examine whether students endorsed the reasons for not engaging in contract cheating differently, as represented by each factor. The overall ANOVA was significant, and all factor means were significantly different from each other (*F*[4,4812] = 886.76, *p* < 0.001, ${}_{\text{p}}^{2}$ = 0.424). As can be seen in Table 2, Morality and Norms was the reason most endorsed by students for not contract cheating, closely followed by Motivation for Learning, with Lack of Opportunity receiving the lowest endorsement.

### Stage Two: Pearson’s Correlation and Multiple Regression Analyses

Bivariate Pearson’s correlations were calculated for the five factors and the psychological measures (see Table 2). Nearly all the correlations between the factors and measures returned statistically significant results with α = 0.001. This is partly attributable to the large sample size. Thus, to conduct more meaningful analyses that extend beyond the original hypotheses multiple linear regressions were calculated.

Five multiple linear regression analyses were conducted to determine which psychological individual differences predicted each factor of the RNCC scale. Due to the distinctly different sample sizes between males (*n* = 120) and females (*n* = 1053), gender was entered into the five regression analyses at the first step followed by the psychological individual differences variables at second step. *Not specified* and missing responses to the demographic question on gender were excluded from the analyses. The multicollinearity assumption for regression was satisfied (all VIFs < 3). The results from the multiple regression analyses are presented in Table 3. No suppressor effects were identified.

**TABLE 3:** Results from the multiple linear regression analyses for the five factors.

	***B* [95% CI]**	**β**	***t***	***sr*^2^**	***R***	***R*^2^**	***ΔR^2^***
**Factor 1: Fear of detection and punishment**							
Step 1					0.002	0.000	0.000
Gender	−0.008 [−0.232, 0.216]	–0.002	–0.07	0.000			
Step 2					0.250	0.063	0.063
Gender	0.070 [−0.157, 0.297]	0.018	0.60	0.000			
Machiavellianism	0.072 [0.018, 0.125]	0.110^∗^	2.61	0.005			
Narcissism	0.080 [0.034, 0.126]	0.128^∗∗^	3.40	0.009			
Psychopathy	−0.039 [−0.098, 0.020]	–0.049	–1.30	0.001			
Self-control	0.024 [−0.117, 0.164]	0.014	0.33	0.000			
Competence satisfaction	−0.043 [−0.119, 0.033]	–0.042	–1.10	0.001			
Competence frustration	0.059 [−0.005, 0.124]	0.067	1.80	0.003			
Consistency of interest	0.095 [0.008, 0.184]	0.081^∗^	2.14	0.004			
Perseverance of effort	0.046 [−0.077, 0.169]	0.028	0.73	0.000			
**Factor 2: Self-efficacy and mistrust**							
Step 1					0.024	0.001	0.001
Gender	−0.098 [−0.328, 0.133]	–0.024	–0.829	0.000			
Step 2					0.295	0.087	0.086
Gender	0.067 [−0.164, 0.298]	0.017	0.57	0.000			
Machiavellianism	0.021 [−0.034, 0.075]	0.031	0.74	0.000			
Narcissism	0.071 [0.024, 0.118]	0.111^∗∗^	2.98	0.007			
Psychopathy	0.074 [0.014, 0.134]	0.090^∗∗^	2.42	0.005			
Self-control	0.181 [0.37, 0.324]	0.104^∗∗^	2.78	0.005			
Competence satisfaction	0.186 [0.109, 0.264]	0.177^∗∗^	4.72	0.017			
Competence frustration	0.030 [−0.035, 0.096]	0.033	0.91	0.001			
Consistency of interest	0.091 [0.001, 0.180]	0.074^∗^	1.98	0.003			
Perseverance of effort	0.152 [0.028, 0.278]	0.090^∗^	2.41	0.004			
**Factor 3: Morality and norms**							
Step 1					0.172	0.029	0.029
Gender	0.531 [0.356, 0.706]	0.172^∗∗^	5.96	0.030			
Step 2					0.345	0.119	0.090
Gender	0.375 [0.201, 0.550]	0.121^∗∗^	4.22	0.013			
Machiavellianism	−0.064 [−0.105, -0.023]	−0.125^∗^	–3.04	0.007			
Narcissism	0.050 [0.015, 0.086]	0.103^∗^	2.80	0.006			
Psychopathy	−0.091 [−0.136, −0.046]	–0.144^∗∗^	–3.93	0.012			
Self-control	0.170 [0.062, 0.278]	0.127^∗^	3.08	0.007			
Competence satisfaction	0.036 [−0.023, 0.094]	0.044	1.20	0.001			
Competence frustration	0.039 [−0.010, 0.089]	0.056	1.56	0.002			
Consistency of interest	0.078 [0.010, 0.145]	0.082^∗^	2.25	0.004			
Perseverance of effort	0.157 [0.062, 0.251]	0.120^∗^	3.26	0.008			
**Factor 4: Lack of opportunity**							
Step 1					0.055	0.003	0.003
Gender	−0.118 [−0.242, 0.005]	–0.055	–1.88	0.003			
Step 2					0.280	0.079	0.076
Gender	−0.036 [−0.160, 0.088]	–0.017	–0.57	0.000			
Machiavellianism	0.008 [−0.022, 0.037]	0.022	0.52	0.000			
Narcissism	−0.006 [−0.031, 0.019]	–0.017	–0.46	0.000			
Psychopathy	0.049 [0.017, 0.081]	0.111^∗^	2.97	0.007			
Self-control	−0.102 [−0.179, −0.025]	−0.110^∗^	–2.60	0.005			
Competence satisfaction	−0.024 [−0.066, 0.017]	–0.043	–1.14	0.001			
Competence frustration	0.033 [−0.002, 0.068]	0.067	1.82	0.003			
Consistency of interest	0.057 [0.008, 0.105]	0.086^∗^	2.31	0.004			
Perseverance of effort	0.039 [−0.028, 0.106]	0.043	1.14	0.001			
**Factor 5: Motivation for learning**							
Step 1					0.070	0.005	0.005
Gender	0.260 [0.047, 0.472]	0.070^∗^	2.93	0.005			
Step 2					0.324	0.105	0.100
Gender	0.095 [−0.116, 0.307]	0.026	0.88	0.001			
Machiavellianism	-0.071 [−0.121, −0.021]	−0.115^∗^	–2.77	0.006			
Narcissism	−0.025 [−0.068, 0.018]	–0.043	–1.16	0.001			
Psychopathy	−0.022 [−0.688, −0.018]	–0.030	–0.82	0.000			
Self-control	0.135 [0.004, 0.267]	0.085^∗^	2.03	0.003			
Competence satisfaction	0.103 [0.032, 0.174]	0.106^∗^	2.85	0.006			
Competence frustration	0.059 [−0.001, 0.119]	0.069	1.92	0.003			
Consistency of interest	−0.024 [−0.106, 0.058]	–0.021	–0.57	0.000			
Perseverance of effort	0.185 [0.071, 0.300]	0.118^∗∗^	3.18	0.008			

*CI, confidence intervals, ^∗^*p* < 0.05, ^∗∗^*p* ≤ 0.001. Gender was coded as male = 1, female = 2.*

Gender was found to be a significant predictor of Morality and Norms and Motivation for Learning at the first step of the regression analyses. However, gender was only significant at the second step for Morality and Norms. These results will be discussed in more detail below along with the other significant predictors for both of these factors.

The regression analysis for Fear of Detection and Punishment accounted for 5.5% of the variance in this factor (*F*[9,1163] = 11.43, *p* < 0.001), and identified three significant predictors from the nine variables, these were: Machiavellianism, narcissism, and consistency of interest. These variables were all positive predictors of Fear of Detection and Punishment, suggesting that students who scored highly on Machiavellianism, narcissism, and consistency of interest are more likely to report fear of detection and punishment as a reason why they do not engage in contract cheating.

The second regression analysis on Self-Efficacy and Mistrust accounted for 8.7% of the variance (*F*[9,1163] = 16.87, *p* < 0.001]), and identified narcissism, psychopathy, self-control, competence satisfaction, consistency of interest, and perseverance of effort as positive predictors of Self-Efficacy and Mistrust. This implies that students who scored highly on these psychological measures were more likely to report reasons relating to self-efficacy and mistrust as contributing to why they do not engage in contract cheating.

The regression analysis on Morality and Norms identified gender as a significant predictor at step one (*p* < 0.001) as well as step two (discussed below). Step two of the regression analysis accounted for 11.9% of the variance (*F*[9,1163] = 13.64, *p* < 0.001), and found gender and all the psychological variables, excluding both competence subscales, were significant predictors. Machiavellianism and psychopathy were both negative predictors of Morality and Norms, suggesting that students who scored highly on these personality traits were less likely to report morality and norms as contributing to why they do not engage in contract cheating. Narcissism, self-control and both grit subscales (consistency of interest and perseverance of effort) positively predicted Morality and Norms, implying that students who score highly in these traits are more likely to report morality and norms as a contributing reason to why they do not engage in contract cheating. Interestingly, the inclusion of gender in the regression resulted in narcissism being a significant predictor despite it not being a significant correlate.

The regression analysis on Lack of Opportunity accounted for 7.9% of the variance (*F*[9,1163] = 4.38, *p* < 0.001), and identified three significant predictors, these are: psychopathy, self-control, and consistency of interest. Self-control negatively predicted Lack of Opportunity, suggesting that students with high self-control were less likely to report a lack of available opportunity as a reason for not engaging in contract cheating. Psychopathy and consistency of interest were positive predictors of Lack of Opportunity. This implies students who scored highly on these measures were likely to report a lack of opportunity as a reason they do not engage in contract cheating.

The final regression analysis, conducted on Motivation for Learning, identified gender as a significant predictor (*p* = 0.002) at the first step, however, gender was not significant at the second step of the analysis. This regression accounted for 10.5% of the variance (*F*[9,1163] = 17.36, *p* < 0.001), and four significant predictors were identified at the second step, these were: Machiavellianism, self-control, competence satisfaction, and perseverance of effort. Machiavellianism was a negative predictor of Motivation for Learning, suggesting students who scored highly on this trait were less likely to report motivation for learning as a contributing reason for why they do not engage in contract cheating. Self-control, competence satisfaction, and perseverance of effort were all positive predictors of Motivation for Learning. This implies students with high self-control, satisfaction of the need for competence, and perseverance of effort were more likely to report not engaging in contract cheating due to reasons relating to their motivation for learning.

In summary, at least one of the Dark Triad subscales was a significant predictor of each of the five factors, with each trait predictive of three factors. The Brief Self-Control Scale was a significant predictor of four of the five factors. Satisfaction of need for competence was found to be predictive of two of the factors, whilst frustration of competence was not a significant predictor of any. The Short Grit Scale was a significant predictor of all five factors.

## Discussion

Previous research demonstrates that around 97% of university students do *not* engage in contract cheating (Curtis and Clare, 2017; Newton, 2018). This exploratory study tested four hypotheses to examine how criminological theories and psychological constructs influence the reasons why the majority of students never engage in this form of academic misconduct.

The first hypothesis predicted that self-control would have a negative relationship with reasons relating to a lack of opportunity for not engaging in contract cheating. Based on the results from the fourth regression, this was supported. Students who had high self-control were found to be unlikely to engage in contract cheating, even when the opportunity was available to them. Conversely, students with low self-control, who reported not engaging in contract cheating when the opportunity was not present, may do so if the opportunity was available. This finding provides support for the use of the General Theory of Crime and routine activity theory and the rational choice perspective as theoretical explanations for why students do not engage in contract cheating.

The second hypothesis predicted that the psychological constructs of self-control, satisfaction of students’ need for competence, and grit would be positively related to reasons connected to student motivation for learning. This was partially supported by the results from regression five. High self-control, competence satisfaction, and perseverance of effort were all significant predictors of Motivation for Learning. Interestingly, consistency of interest was not a significant predictor of Motivation for Learning. Wolters and Hussain (2015) found similar results, where consistency of interest was not a significant predictor of their two measures of academic motivation. Credé et al. (2017) found that only perseverance of effort explained the incremental variance of academic performance, whilst consistency of interest explained almost none. Consistent with this, Amigud and Lancaster’s (2019) recent work, looking at student discourse on Twitter, found that perseverance, or a lack thereof, was the most stated reason that students gave for seeking to outsource assignment work.

The third hypothesis predicted that the Dark Triad personality traits would have a negative relationship with reasons connected to morals and norms for not engaging in contract cheating. This hypothesis was partially supported, based on the results from the third regression. Machiavellianism and psychopathy both negatively predicted Morals and Norms, however, narcissism positively predicted the factor. Narcissism has been found to have a weak relationship with normative values (Jonason et al., 2018) which may explain this finding. Jonason et al. (2018) note that individuals with narcissistic traits have a “more ‘positive’ approach to dealing with others” (p. 6) when compared to those with strong Machiavellian or psychopathic traits.

Finally, the fourth hypothesis predicted that Dark Triad personality traits would have a positive relationship with fear of detection and punishment as a reason for not engaging in contract cheating. This hypothesis was also partially supported, based on the results from the first regression analysis. Significant results were found for Machiavellianism and narcissism, but not for psychopathy. Machiavellians and narcissists tend to be sensitive to the environmental contingencies that may result from their behaviour (Jones and Paulhus, 2017). In contrast, psychopathy has been associated with a disregard for consequences, such as punishment (Viding et al., 2014), which may explain this finding. Additionally, individuals with psychopathic traits are believed to have lower self-control compared to the other Dark Triad traits (Jones and Paulhus, 2011). Applying the General Theory of Crime (Gottfredson and Hirschi, 1990) we can infer that psychopathic individuals are more likely to seek immediate gratification and have a greater susceptibility to temptation, regardless of consequences. Thus, these individuals appear to be more likely to engage in contract cheating compared to Machiavellians and narcissists.

The results of this study support the use of the General Theory of Crime and opportunity-based criminological theories to explain why the majority of students do not engage in contract cheating. These findings are consistent with the literature on the General Theory of Crime and academic misconduct (see Cochran et al., 1998; Curtis et al., 2018). Additionally, although this study examined why students do *not* engage in contract cheating, the results are also, inversely, consistent with the literature on why students do engage in academic misconduct (Franklyn-Stokes and Newstead, 1995; Park, 2003; Devlin and Gray, 2007; Ogilvie and Stewart, 2010; Walker and Townley, 2012; Rigby et al., 2015). Recent research by Bretag et al. (2018a) found that students who engaged in cheating behaviours believe more opportunities are available. Combining this with the current study, if these opportunities can be removed, and self-control can be strengthened within the student population (see Oaten and Cheng, 2006; Muraven, 2010), then this may reduce student engagement in contract cheating.

It is worth noting that there were many additional findings in this study beyond the expectations of the proposed hypotheses. These findings include: consistency of interest positively predicting Fear of Detection and Punishment; narcissism, psychopathy, self-control, competence satisfaction, consistency of interest, and perseverance all positively predictive Self-Efficacy and Mistrust; self-control, consistency of interest, and perseverance of effort positively predicting Morality and Norms; psychopathy and consistency of interest positively predicting Lack of Opportunity; and Machiavellianism negatively predictive Motivation for Learning. Theorising on the relationships identified above is beyond the scope of this paper but may be of interest in future research.

### Practical Implications

Despite being cross-sectional and correlational, this study presents some potential practical implications. Although it is difficult to change innate psychological traits, it is possible to influence the reasons why students do not engage in contract cheating. Tertiary institutions may use the reasons why students do not engage in this behaviour to help guide and shape academic policies on contract cheating and, potentially, other forms of academic misconduct. The results from this study may also be used to inform assessment design. Baird and Clare (2017) suggest that the opportunity structure for academic misconduct can be disrupted by implementing appropriate assessment designs. Bretag et al. (2018b) have also been investigating how assessment design may influence student engagement in contract cheating. The current study suggests that it may be possible to deter students from engaging in contract cheating by emphasising the different reasons identified here, including: motivation to learn, morals and norms, and risk of detection and punishment. As mentioned previously, by helping students develop their self-control and interrupting the opportunity structure, such as through assessment design, tertiary institutions can further reduce student engagement in contract cheating (e.g., Baird and Clare, 2017). Other practical guidance can be found in Tertiary Education Quality and Standards Agency’s (Tertiary Education Quality and Standards Agency [TEQSA], 2017) Good Practice Note on contract cheating.

### Limitations

There are a number of limitations present in the current study. First, this is a cross-sectional, correlational study meaning it is not possible to draw cause-and-effect conclusions about the findings. Second, it is possible that some students’ reason for not engaging in contract cheating is that they were unaware that this cheating option existed before they were provided with the definition of contract cheating in our questionnaire. For such students, their response to all items of the RNCC measure would logically be “Not a reason at all,” because their reason for not cheating was unlisted. Although the data suggests little evidence of this response pattern, such responses would create error variance that may have weakened the relationships observed in this study. Future research into students’ reasons for not cheating should ask the students whether they are aware of the form of cheating under examination. Third, more recent literature on contract cheating has defined a range of outsourcing behaviours by students as variants on contract cheating (e.g., Bretag et al., 2018a, b). Our definition was limited to what may be described as *commercial contract cheating*. It is possible that students who did not use commercial contract cheating services have engaged in other forms of similar behaviour such as obtaining ghost-written assignments from family members. Fourth, there is the potential for social desirability bias to be present in the current study, caused by participants responding in a manner that they believe is socially appropriate (Tourangeau and Yan, 2007). However, given that the prevalence of engagement in contract cheating identified in this study was comparable to other similar research (around 2%; e.g., Curtis and Vardanega, 2016; Curtis and Clare, 2017; Newton, 2018), it can be expected that underreporting in this instance was, at least, no worse than in other designs. Nonetheless, future research would benefit from the inclusion of a measure of social desirability. Fifth, there has been some critical analysis of the construct of grit since the development and undertaking of this study (Credé et al., 2017). This critique has suggested that grit may be a component of conscientiousness (Credé et al., 2017). Consequently, future research may benefit from examining conscientiousness in place of, or even in conjunction with, grit.

Finally, caution must be used when interpreting the results due to potential sampling biases, including a substantial gender imbalance and sampling methods (resulting in a large response from the Western Australian University, psychology major students, and the use of social media to attract respondents). Just over 10% of the total sample identified as male, this gender imbalance may have influenced the findings of this study. Despite suggestions that males are more likely to engage in academic misconduct (see Selwyn, 2008; Hensley et al., 2013; Kuntz and Butler, 2014), there is inconclusive support for this relationship in the literature (see Underwood and Szabo, 2003; Kisamore et al., 2007; Curtis and Vardanega, 2016). However, males, on average, have been found to score higher on measures of the Dark Triad traits compared with females (Paulhus and Williams, 2002; Jonason and Webster, 2010; Roeser et al., 2016). In contrast, females tend to score higher in grit than males (Kannangara et al., 2018). Additionally, Ivert et al. (2018) suggest that females have stronger moral values than males, this difference is likely to have resulted in the significant gender effects in the regression on Morality and Norms. In order to address the limitation of gender imbalance in this study, gender was controlled for in the regressions. However, future research would benefit from more equal sample comparisons between genders, as this would add to the argument on whether males are more likely to engage in academic misconduct as well as addressing this limitation in the current study. Furthermore, the use of SurveyCircle and social media is likely to have introduced further biases, including potentially exacerbating the gender imbalance, which similarly occurred in Foltýnek and Králíková’s (2018) research. Thus, future research may benefit from employing additional sampling techniques in order to address this limitation.

### Future Directions

An obvious future direction from this study is to examine the inverse, why *do* students engage in contract cheating, using similar measures. This would enable a comparison of how reasoning differs between those who engage in this behaviour compared with those who refrain. Future research in this area may also benefit from the inclusion of other measures, such as: academic self-efficacy and autonomy. Academic self-efficacy refers to an individual’s confidence in their own ability to perform academic tasks (Gore, 2006). Ogilvie and Stewart (2010) found that low academic self-efficacy is associated with engagement in academic misconduct. Additionally, autonomy (having control over one’s own choices and behaviours, Longo et al., 2016), has been associated with student motivation and self-efficacy in goal-setting (Vieira and Grantham, 2011). Perceptions of autonomy were found to help students feel more confident in their ability to complete tasks, resulting in an increased commitment to more challenging goals (Vieira and Grantham, 2011). In the context of contract cheating, both autonomy and academic self-efficacy can potentially offer a greater understanding of why students do not engage in contract cheating. Students who feel capable of performing academic tasks and are committed to challenging goals are potentially less likely to engage in contract cheating behaviours.

Detection tools are also emerging in this field, for example Turnitin’s (2018) Authorship Investigation software, that may allow contract cheating to be examined in new ways. This software has been touted to be able to detect possible cases of contract cheating by developing a database based on each students’ independent writing style and document metadata (Turnitin, 2018). Future research may benefit from examining how these new tools affect student engagement in contract cheating and how they may alter the reasons why students do not engage in contract cheating.

## Conclusion

Contract cheating is a serious issue facing tertiary institutions, but happens at a relatively low frequency across students. This study sought to address a significant gap in the literature by examining the reasons why the majority of students do *not* engage in contract cheating (and even students who do cheat this way do not do it all the time) and how individual differences can influence these reasons and variation across assessment items. The results from this study supported the theoretical explanations offered by the criminological theories of self-control and opportunity, and suggested that the main reasons students refrain from engaging in contract cheating is due to their sense of morals, perception of norms, and their motivation to learn. The individual differences measured in this study, particularly self-control and the Dark Triad personality traits, were predictive of student reasoning. Tertiary institutions may be able to decrease student engagement in contract cheating by influencing these factors, such as by encouraging students to develop their self-control and by interrupting the opportunity structure that enables contract cheating. In addition, our findings suggest that emphasising learning goals, ethics, and the low normative endorsement of contract cheating may bolster students’ rationales for not cheating. By reducing engagement in this form of cheating, tertiary institutions can bolster the validity of assessments in higher education to evaluate student learning. Future research should aim to address the limitations discussed above and build upon the findings of this study.

## Data Availability Statement

The datasets generated for this study are available on request to the corresponding author.

## Ethics Statement

The studies involving human participants were reviewed and approved by the Murdoch University Human Research Ethics Committee. The patients/participants provided their informed consent to participate in this study.

## Author Contributions

KR, GC, and JC contributed to the conception and design of the study. KR conducted the study under the supervision of GC and JC, and conducted the statistical analysis with the assistance of GC. KR wrote the first draft of the manuscript, with assistance provided by GC and JC. All authors contributed to the manuscript revision, and read and approved the submitted version.

## Conflict of Interest

The authors declare that the research was conducted in the absence of any commercial or financial relationships that could be construed as a potential conflict of interest.

## Footnotes

1 The Western Australian University’s online management system for student participation in psychology studies. Credit from this system is a requirement in three units for undergraduate psychology students.

2 Participants were rewarded with a choice of: Research Participation Portal credit (0.5) from the Western Australian University, SurveyCircle points, or the chance to win a $50AUD Amazon gift card.

3 The Trust Propensity Scale (Mayer and Davis, 1999) was also included in the study. However, the results from this measure did not add anything of value to the results and has been excluded here.

## Appendix

Contract cheating is the process of paying another individual to write an assignment and claiming the work as your own.

Have you ever engaged in contract cheating?

Yes

No

For each item below, please indicate the extent to which this is a reason that you **do not** engage in contract cheating using the following scale:

1 = Not a reason at all

2 = This contributes slightly to the reason I do not

3 = This contributes somewhat to the reason I do not

4 = This contributes a lot to the reason I do not

5 = This is the main reason I do not

(1) I think it is wrong or immoral

(2) I am afraid of getting caught

(3) I am afraid of being punished

(4) I’m afraid that being caught would have long-term career implications

(5) I feel I’d only be cheating myself because I am studying to learn

(6) I am studying to learn rather than to get a qualification/degree

(7) I feel I could do better than someone I paid

(8) I can’t afford to pay someone to write assignments for me

(9) I would not know where to find someone to write an assignment for me

(10) I would feel shame, guilt or remorse

(11) I don’t think other students like me would pay someone to write their assignment

(12) I have not completed any assignments, so I have not had the chance or need

(13) I could not find someone to write on the specific assignment topic

(14) I don’t care enough about grades to want to cheat to do better

(15) I manage my time well and don’t have to resort to cheating

(16) I don’t trust the other people to do my assignment well

(17) I don’t trust people to do my assignment on time

(18) I want the sense of achievement from doing work myself

(19) I’m afraid that if others found out I would be judged negatively or ridiculed

(20) I wouldn’t do it because I would not want others to do it

(21) I am worried I would be caught by plagiarism software like Turnitin or Urkund
